# The South Australian ^177^Lu-DOTATATE peptide receptor radionuclide therapy service: an 11-year review of toxicity, health-related quality of life, and survival

**DOI:** 10.1016/j.esmogo.2025.100146

**Published:** 2025-03-04

**Authors:** L.M. Altus, J.C. Forster, J. Mercurio, M. Kitchener, N. Corsini, M. Nenke, T. Price, D. Patel, R. Chew, D. Moffat, S. Unger, G. Cehic

**Affiliations:** 1 Department of Nuclear Medicine, The Queen Elizabeth Hospital, Woodville South, Australia; 2 Adelaide Medical School, University of Adelaide, Adelaide, Australia; 3 Medical Physics and Radiation Safety, South Australia Medical Imaging, Adelaide, Australia; 4 Department of Physics, University of Adelaide, Adelaide, Australia; 5 University of South Australia, Clinical & Health Sciences (Rosemary Bryant AO Research Centre), South Australia, Adelaide, Australia; 6 Endocrine Department, Central Adelaide Local Health Network, Adelaide, Australia; 7 Department of Medical Oncology, The Queen Elizabeth Hospital, Woodville South, Australia; 8 Department of Medical Oncology, Northern Adelaide Local Health Network, Elizabeth Vale, Australia; 9 Department of Anatomical Pathology, SA Pathology, Flinders Medical Centre, Bedford Park, Australia; 10 College of Medicine and Public Health, Flinders University, Bedford Park, Australia; 11 University of South Australia, Allied Health & Human Performance, South Australia, Adelaide, Australia

**Keywords:** peptide receptor radionuclide therapy (PRRT), neuroendocrine neoplasms/tumours (NENs/NETs), [^177^Lu]-DOTA-octreotate (^177^Lu-DOTATATE), health-related quality of life (hr-QoL), radioligand therapy (RLT), theranostics

## Abstract

**Background:**

The aim of this study was to audit clinical and patient-reported outcomes of patients treated with [^177^Lu]-DOTA-octreotate (^177^Lu-DOTATATE) at a single Australian centre over an 11-year period.

**Methods:**

A retrospective analysis of 189 patients with metastatic or locally advanced neuroendocrine neoplasms or tumours (NENs/NETs) or other somatostatin receptor-positive tumour treated with ^177^Lu-DOTATATE at The Queen Elizabeth Hospital from 2011 to 2022 was carried out. Toxicity, radiological response, health-related quality of life (hr-QoL), and overall survival (OS) were summarised.

**Results:**

Gastroenteropancreatic (NET) origin accounted for 75% of cases. The median age of diagnosis was 59 years and median age at first cycle of ^177^Lu-DOTATATE at The Queen Elizabeth Hospital was 64.7 years (range 16.5-92.6 years). Of the 182 patients who commenced induction peptide receptor radionuclide therapy (iPRRT), 143 (79%) completed four or five induction cycles. There were 52 patients who received retreatment PRRT (rPRRT). When radiological response was assessed following completion of a full iPRRT course (≥four cycles, 131 patients), radiologically stable disease was seen in 76% of patients. A completed hr-QoL questionnaire was available for 760 cycles (92%). Significant improvements were found across multiple domains after iPRRT. Eight patients (4.2%) developed a haematological malignancy. Renal function declined at long-term but not short-term follow-up. Median duration of follow-up was 37.6 months (range 0.0-134.6 months) with a median OS from first cycle of PRRT 48.4 months (95% confidence interval 42.5-57.3 months).

**Conclusion:**

Patients with advanced NEN/NET or other somatostatin receptor-positive malignancies treated with ^177^Lu-DOTATATE at our centre had improved hr-QoL following iPRRT, with comparable toxicity and OS to other centres.

## Highlights

• We report on 189 patients who received ^177^Lu-DOTATATE at a single South Australian centre over 11 years.
• Median overall survival was 48.4 months (95% CI 42.5-57.3 months).
• Significant improvements were found across multiple hr-QOL domains following induction therapy.
• Haematological malignancy occurred in 8 (4.2%) patients.

## Introduction

Theranostics is an established pillar of cancer care and is increasingly used as a targeted treatment option for selected cancers. For patients with progressive metastatic gastroenteropancreatic (GEP) neuroendocrine neoplasms or tumours (NENs or NETs), peptide receptor radionuclide therapy (PRRT) with [^177^Lu]-DOTA-octreotate (^177^Lu-DOTATATE) has proven safety and efficacy in extending progression-free survival and improving health-related quality of life (hr-QoL).^1^, ^2^, ^3^, ^4^, ^5^ The role for PRRT in the treatment of other somatostatin receptor (SSTR)-positive neoplasms, including bronchial carcinoid,^6^ paraganglioma, and phaeochromocytoma,^7^ is also increasingly recognised.

The South Australian PRRT service, based at The Queen Elizabeth Hospital (TQEH) in Adelaide was established in 2011 and provides comprehensive care for patients with advanced SSTR-expressing tumours. Before the development of a dedicated radionuclide service, a small number of eligible patients were treated at interstate PRRT centres. The first cycle of ^177^Lu-DOTATATE was administered at TQEH in April 2011. As of 31 December 2024, the service has treated 231 individual patients and administered just over 1000 cycles.

As the sole PRRT centre for the central corridor of Australia, which includes South Australia, Northern Territory, and rural New South Wales, the geographical spread of our patients has created major challenges for equitable access. An area greater than 2 300 000 km^2^ is serviced, equivalent to the combined areas of France, Spain, Sweden, Norway, and Germany. Some patients have travelled over 2500 km for treatment in Adelaide. Approximately 30% of our patients reside in rural and remote locations.^8^

Demographic, clinical, pathological, and hr-QoL data have been collected as standard of care. We present the first detailed audit of our patient cohort since inception of the service, summarising outcomes including toxicity, hr-QoL, and overall survival (OS). Our intention is to share our centre’s real-world experience and add to the body of literature in clinical theranostics.

## Methods

### Eligibility and access

Patients eligible for referral for induction PRRT (iPRRT) or retreatment PRRT (rPRRT) were discussed at the statewide NET multidisciplinary tumour board meeting (NET MDT). Following referral, patients underwent consultation with the TQEH PRRT team, consisting of a nuclear medicine physician, physician fellow, and dedicated specialist oncology NET nurse. To confirm eligibility, renal perfusion study and 3-time-point glomerular filtration rate (GFR) using ^99^^m^Tc-diethylenetriaminepentaacetic acid (DTPA), complete blood examination, electrolytes, and renal function [estimated GFR (eGFR)], liver function tests, and disease-specific biomarkers were carried out.

Selection criteria for PRRT:

• Histologically confirmed NEN/NET or neoplasm with neuroendocrine differentiation
• Locally advanced/inoperable/metastatic disease
• Progression on at least first-line therapy (as demonstrated radiologically, biochemically, and/or symptomatically)
• Adequate SSTR expression on [^68^Ga]-DOTATATE positron emission tomography (PET), specifically a modified Krenning scale ≥2
• Absence of 2-deoxy-2-[^18^F]fluoro-d-glucose (FDG) PET incongruent disease if carried out
• GFR >40 ml/min based on DTPA renal perfusion study
• Sufficient bone marrow reserve
• Adequate functional status
• For those referred for retreatment, previous response to iPRRT

Given the palliative nature of this treatment, some exceptions to the selection criteria were permissible by the MDT in accordance with other PRRT guidelines adopting a personalised approach. For example, PRRT was given as first-line salvage therapy when tumour and symptom burden was high, or for other SSTR-expressing tumours where PRRT is less established.

### PRRT protocol and procedure

Patients were planned for an initial iPRRT course of four cycles of approximately 7.4 GBq of ^177^Lu-DOTATATE at 8-week intervals. Those undergoing rPRRT were planned for up to four cycles. Those with higher-grade disease, high tumour burden, or at the discretion of the referrer/MDT were considered for concurrent single-agent chemotherapy (intravenous 5-fluorouracil or oral capecitabine) or combination oral capecitabine/temozolomide (CAPTEM), designated as PRCRT.

Long-acting somatostatin analogues (SSAs) were held at least 3 weeks before radionuclide therapy or tailored to symptom burden. Patients classified at increased risk of carcinoid crisis were admitted to hospital for close monitoring up to 48 h before and after treatment, with administration of short-acting octreotide as per our hospital guideline.^9^

European Organisation for Research and Trials Collaboration (EORTC) quality-of-life questionnaires (QLQ) to assess hr-QoL were completed at multiple time points including the initial consultation, each cycle of ^177^Lu-DOTATATE, and at follow-up consultation at 3 months. From 2011 to 2014, the QLQ Core 30 (QLQ-C30) was used,^10^^,^^11^ with the addition of the QLQ–GINET21 companion questionnaire^12^ from 2015 onwards. Responses were used to evaluate disease-related symptom burden to inform clinical management decisions.

For each cycle, patients received anti-emetics (ondansetron or equivalent, and dexamethasone) and an infusion of amino acids (25 g lysine and 25 g arginine per litre) over 4 h for renal protection, commencing 30 min before the ^177^Lu-DOTATATE injection. ^177^Lu-DOTATATE was sourced from local or international suppliers pending availability. It was only accessible through the Special Access Scheme of the Therapeutic Goods Administration of Australia and remains unfunded in Australia at the time of publication.

Post-therapy whole body planar imaging was carried out at 4 h, with patients returning at 24 h for triple-field single photon emission computed tomography (SPECT/CT) imaging. Dexamethasone was continued for 2 days following therapy, with additional antiemetics prescribed as required. Haematological, renal, and hepatic toxicity were closely monitored. All patients received a personalised schedule detailing the timing of post-therapy medications, SSA injections, blood tests, and appointments.

After completion of iPRRT or rPRRT, patients returned for follow-up review at 3 months. Haematological, renal, and liver function blood tests; biomarkers; hr-QoL scores; and restaging imaging were used to assess treatment response and toxicity. Data were stored in the online Research Electronic Data CApture Platform (REDCap),^13^^,^^14^ sponsored by the Central Adelaide Local Health Network (CALHN) research and ethics unit.

### Clinical audit

A retrospective clinical audit was conducted of our patients treated with ^177^Lu-DOTATATE between 2011 and 2022. Data were extracted from the REDCap database with approval from the CALHN human research ethics committee.

The audit included all induction and retreatment courses of ^177^Lu-DOTATATE that met the following criteria: all cycles in the course occurred before 31 December 2022; no further cycles were planned for that course; and if there was a restaging scan following the course, it was not after 31 December 2022. All patients who received at least one such course were included in the audit.

### Assessment and outcomes in audit

Radiographic response was determined by a radiologist or investigator using Response Evaluation Criteria in Solid Tumours version 1.1 (RECIST 1.1), comparing pretreatment imaging to the restaging scan carried out 2-6 months after the last cycle in a course.

Hr-QoL questionnaire responses were scored according to the EORTC manual into a standardised 0-100 scale. Overall scores were compared from cycle 1 of iPRRT to the posttreatment follow-up review if available, or else to the last completed iPRRT cycle.

Renal function was reviewed at 3 months after iPRRT. The last available renal and haematological results from either before death or within 6 months of 31 December 2022 (the “census date”) were also reviewed for all patients.

Follow-up and OS were calculated from the day of the first cycle of ^177^Lu-DOTATATE at TQEH to the date of death or the census date. For calculations that required a date of diagnosis, if only a year of diagnosis was available, the date of diagnosis was designated 1 January.

### Statistical analysis

Kaplan–Meier analyses were applied to evaluate median OS. Cox proportional hazards regression was used to identify markers independently associated with OS. All statistically significant markers in univariate analysis were further incorporated into multivariate analysis. One-tailed paired *t*-tests were used to compare mean hr-QoL scores before and after iPRRT. A *P* value of <0.05 was regarded as statistically significant. Statistical analyses were carried out in Python using lifelines (version 0.27.8)^15^ and SciPy (version 1.11.4).^16^

## Results

### Patient and disease characteristics

Between 2011 and 2022, 189 patients met the inclusion criteria for the audit. All patients were Australian residents. Patient and clinical characteristics are summarised in Table 1.

**Table 1:** Baseline characteristics of patients who received ^177^Lu-DOTATATE treatment at TQEH during 2011-2022

Characteristic	Number of patients (% of total cohort)
Total number of patients	189
Gender	
Male	93 (49%)
Female	96 (51%)
Median age at diagnosis	59 years (12-84 years)
Primary site	
*GEP-NET*	141 (75%)
Small bowel (NOS)	52
Pancreas	45
Lower jejunum/ileum	26
Colon/rectum	9
Ovary	4
Renal	2
Appendix	1
Gastric/stomach	1
Duodenum/ampulla/proximal jejunum	1
*Lung*	12 (6%)
Atypical	7
Typical	3
Unknown	2
*Other*	13 (7%)
Paraganglioma	8
Phaeochromocytoma	2
Neuroblastoma	1
Neuroendocrine carcinoma of the breast	1
Myoepithelial carcinoma	1
*Unknown*	23 (12%)
GEP-NET grade (WHO 2017 criteria) (*n* = 141)^a^	
Grade 1	41 (29%)
Grade 2	59 (42%)
Grade 3—well differentiated	4 (3%)
Unknown/unable to determined	37 (26%)
Sites of metastasis	
Lung	18 (10%)
Liver	160 (85%)
Bone	78 (41%)
Nodal	97 (51%)
Soft tissue	39 (21%)
Other site	25 (13%)
Disease-specific treatments^b^ before first ^177^Lu-DOTATATE at TQEH	
Somatostatin analogue	181 (96%)
Surgery	95 (50%)
Chemotherapy	47 (25%)
Radiotherapy—EBRT or SBRT	23 (12%)
Targeted therapy (everolimus/sirolimus)	8 (4%)
Liver directed	9 (5%)
Prior PRRT (^90^Y, ^111^In, or ^177^Lu elsewhere)	7 (4%)
Other	3 (2%)
Cumulative disease specific treatments before ^177^Lu-DOTATATE at TQEH	
1	59 (31%)
2	71 (38%)
3	28 (15%)
4	21 (11%)
≥5	10 (5%)
Other clinical characteristics	
Hormone symptoms present (clinically assessed) (*n* = 176)	96 (55%)
Raised baseline chromogranin A level (*n* = 183)	152 (83%)
Median baseline GFR (*n* = 183)	86.6 ml/min/1.73 m^2^ (40.9-154.4 ml/min/1.73 m^2^)
Median time from diagnosis to first PRRT at any centre	3.1 years (0.2-42.6 years)
Median age at first cycle of ^177^Lu-DOTATATE at TQEH	64.7 years (16.5-92.6 years)

EBRT, external beam radiation therapy; GEP, gastroenteropancreatic; GFR, glomerular filtration rate; NET, neuroendocrine tumour; NOS, not otherwise specified; PRRT, peptide receptor radionuclide therapy; SBRT, stereotactic body radiation therapy; TQEH, The Queen Elizabeth Hospital.

a If more than one pathology sample was available before cycle 1, the more recent grade was used.

b Some patients received multiple of the same line of treatment.

GEP-NET origin accounted for 75% of cases. Small bowel [including not otherwise specified (NOS), jejunum, ileum, duodenum, ampulla] was the most common primary site (41%) followed by pancreas (24%). Grading was available for 104/141 GEP-NET cases, with 96% of these grades 1 and 2. Overall, 16% of grading came directly from review of histology during the MDT meeting.

The most common prior therapy was SSA (96%) followed by surgery (50%). For the eight patients not documented to have received prior SSA therapy, one had NET of unknown primary, one had atypical lung NET, and six had paraganglioma/phaeochromocytoma or neuroblastoma, for which SSA therapy was not approved. A total of seven patients had received prior PRRT; four had received ^177^Lu-DOTATATE at interstate centres and five had received PRRT with other radiopharmaceuticals: three in South Australia, one interstate, and one in the UK. Overall, 31% of patients had received three or more disease-specific treatments before their first cycle of PRRT at TQEH.

Baseline GFR DTPA scan results were available for 183/189 (97%) patients with a median value of 86.6 ml/min (range 40.9-154.4 ml/min). Baseline eGFR results were available for 179 patients who underwent iPRRT, with 24/179 (13%) <60 ml/min/1.73 m^2^ (range 32.0 to >90 ml/min/1.73 m^2^).

### Treatments

The most common reasons for referral for ^177^Lu-DOTATATE were radiological disease progression (92%), biochemical progression (57%), and worsening symptoms (48%). Only 21% of patients were referred for progression on imaging alone, while most had a combination of clinical/biochemical and radiological progression. There were six patients referred for other reasons including consolidation following chemotherapy, high tumour burden, and the presence of severe tricuspid regurgitation from carcinoid heart disease at the time of diagnosis.

Of the 251 courses of radionuclide therapy, 182 were iPRRT, with the remainder rPRRT. Treatment details are summarised in Table 2. In total, 822 cycles of ^177^Lu-DOTATATE were administered during the audit period. The median number of cycles (including both iPRRT and rPRRT courses) administered to a patient at TQEH was 4 (range 1-12).

**Table 2:** Summary of induction and retreatment radionuclide therapy

Total number of cycles given at TQEH	822
Median cumulative administered activity (GBq)	31.0 (4.9-88.7)
Number of iPRRT cycles completed per patient (182 patients)	
1	7 (4%)
2	14 (8%)
3	18 (10%)
4	141 (77%)
5	2 (1%)
Total number of iPRRT cycles	663
Reasons from withdrawing from iPRRT (completing one to three cycles) (39 patients)	
Haematological	13
Medically unfit	9
Patient deceased since last cycle	6
Disease progression	4
Contraindicated (renal)	2
Good response to earlier cycles and decision made to withdraw	2
Contraindicated (liver)	1
Patient refused	1
Bowel obstruction	1
rPRRT (52 patients)	
Number of rPRRT courses	69
Number of patients who had multiple courses of rPRRT	13
Total number of rPRRT cycles	159
Median time (months) between completing iPRRT and returning for rPRRT	24.7 (9.7-85.8)

iPRRT, induction peptide receptor radionuclide therapy; rPRRT, retreatment peptide receptor radionuclide therapy; TQEH, The Queen Elizabeth Hospital.

Of the 182 patients who commenced iPRRT at TQEH, 143 (79%) completed four or five cycles, 18 (10%) completed three cycles, 14 (8%) completed two cycles, and seven (4%) completed only one cycle. For the 39 patients who were unable to complete a full iPRRT course, the leading causes for withdrawal were haematological reasons (13 patients) and being medically unfit to proceed (8 patients).

A total of 52 patients underwent retreatment (rPRRT), including 13 patients who had had multiple rPRRT courses. A total of 159 retreatment cycles were administered with a median of 2 cycles (range 1-8 cycles). The median interval between the last iPRRT cycle and first rPRRT cycle was 24.7 months (range 9.7-85.8 months).

Concurrent chemotherapy was administered with at least 1 cycle of PRRT for 76 patients (40%) and 273/822 cycles (33%). For the GEP-NET patients with a known histological grade, 100% of grade 3, 41% of grade 2, and 32% of grade 1 received at least one cycle of concurrent chemotherapy.

The median administered activity of ^177^Lu-DOTATATE per cycle was 7.7 GBq (range 4.0-9.0 GBq). Some 18% of patients had at least one cycle with intentionally reduced administered activity <7.0 GBq. The median total activity administered to patients was 31.0 GBq (range 4.9-88.7 GBq).

### Response to treatment

#### Radiological response

CT was the imaging modality used for 82% of restaging scans. The median timing of restaging imaging was 3.0 months (range 0.7-9.6 months).

Radiological response to iPRRT was available for 153/182 patients (84%). Of these, 131 patients had received a full iPRRT course (four or more cycles) and 22 patients received a partial iPRRT course. For the patients who received a full iPRRT course, 99 patients (76%) had stable disease (SD), 19 (15%) had partial response (PR), and 13 (10%) progressive disease (PD) (Table 3). For the 22 patients who partially completed induction courses, 68% had SD and the remainder had PD.

Radiological response was available for 47/69 rPRRT courses; 33 courses (70%) had SD, 3 (6%) had PR, and 11 (23%) had PD.

During the audit period, no patients achieved a complete response following either iPRRT or rPRRT.

#### Quality of life

**Figure 1:**
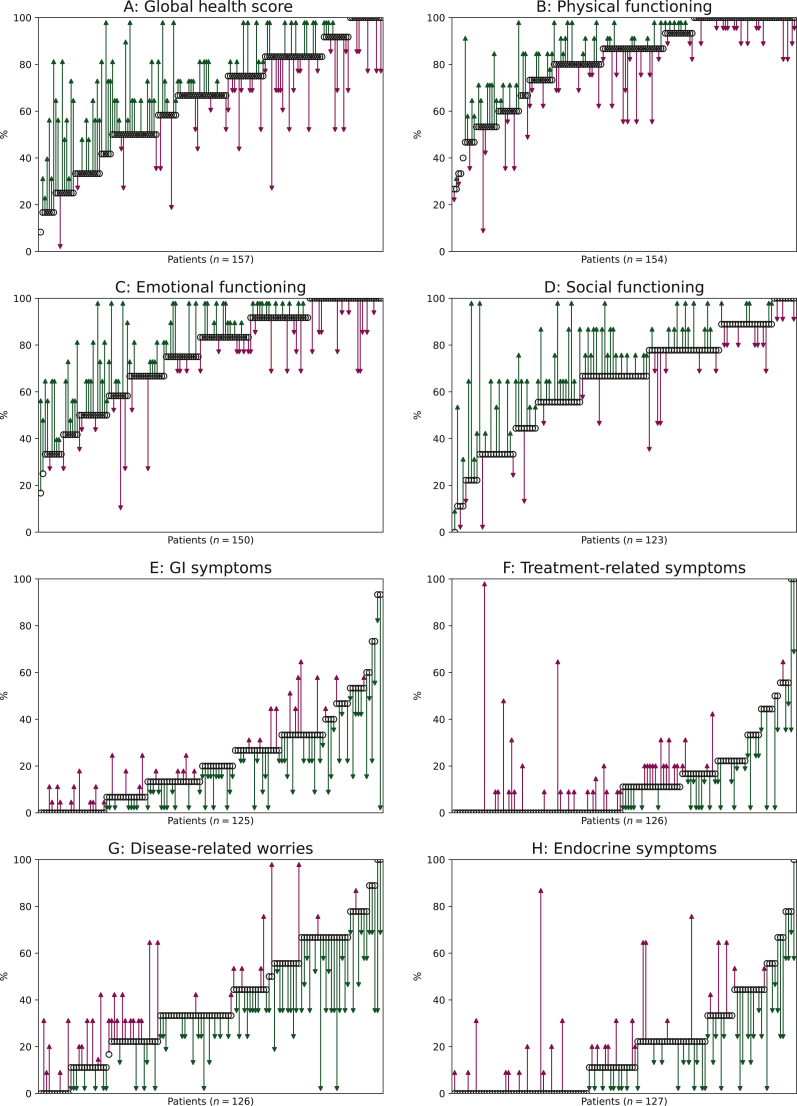
**Change in hr-QoL scores over inductionPRRT.** For each patient, the black circle represents the initial score, and the arrow shows the direction and magnitude of change (green indicates improvement, purple indicates deterioration). GI, gastrointestinal; hr-QoL, health-related quality of life.

Of the 822 ^177^Lu-DOTATATE cycles administered at TQEH, 760 (92%) had a corresponding hr-QoL questionnaire completed. The first 40 patients completed the QLQ-C30 only, until the companion questionnaire was added in 2015. We compared available hr-QOL scores for patients undergoing iPRRT. Figure 1 shows the change in individual patient hr-QoL scores over iPRRT, highlighting at first the heterogeneity of baseline scores across the cohort before commencing ^177^Lu-DOTATATE.

There were significant improvements in global health score, emotional functioning, gastrointestinal symptoms, disease-related worries and social functioning from before to after iPRRT. No significant association was found between change in global health score and radiological response rate or OS.

### Overall survival

The median duration of follow-up was 37.6 months (0.0-134.6 months). At the census date, 110 patients had died (58%). Cause of death was NET-related in 81 patients (74%). Other causes of death were unknown (15%), unrelated (7%), and complication of treatment (5%).

**Figure 2:**
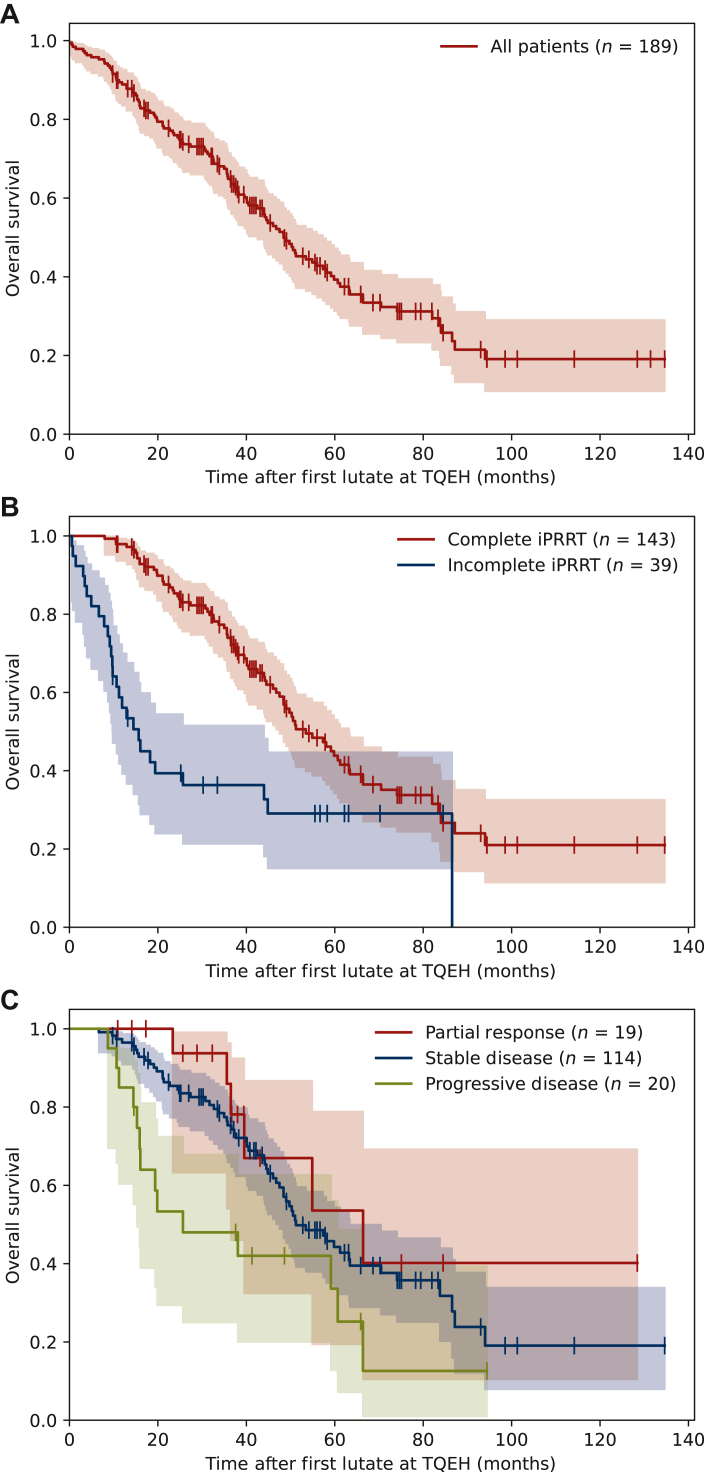
**Overall survival from the first cycle of**^**177**^**Lu-DOTATATE at TQEH.** (A) OS including all patients. The median OS was 48.4 months (95% CI 42.5-57.3 months). (B) OS for complete versus incomplete iPRRT. Complete iPRRT had median 53.5 months (95% CI 47.5-63.2 months) and incomplete iPRRT had median 15.6 months (95% CI 9.7-44.0 months). (C) OS by radiological response following iPRRT. Partial response had median 66.4 months (95% CI 36.5 months to NA), stable disease had median 51.2 months (95% CI 47.5-63.4 months), and progressive disease had median 25.7 months (95% CI 15.3-60.7 months). CI, confidence interval; OS, overall survival; iPRRT, induction peptide receptor radionuclide therapy; TQEH, The Queen Elizabeth Hospital.

The estimated median OS was 48.4 months [95% confidence interval (CI) 42.5-57.3 months] for all patients (Figure 2A). Patients who had a complete iPRRT course of at least four cycles had a significantly greater OS than patients who had an incomplete course of less than four cycles (Figure 2B).

Patients with PD during treatment had significantly worse survival than patients with PR in univariate analysis (Figure 2C). However, this association was not significant when adjusted for iPRRT completion. OS was not significantly associated with tumour primary site or GEP-NET grade.

### Safety and tolerability

^177^Lu-DOTATATE was well tolerated, with 438/822 (53%) cycles completed without any symptoms occurring on the day of therapy. The most common side-effects were minor flushing (25%) and nausea (20%), with other symptoms of dizziness, vomiting, pain, or palpitations each occurring in <2% of cycles. Only one patient required an unplanned admission for carcinoid crisis after PRRT, which was managed with octreotide infusion and the patient was discharged after 24 h.

Haematological results were available for 92% of patients from within the last 6 months of death or the census date. Over the 11-year audit period, 8/189 patients (4.2%) developed a haematological malignancy, with 7 of the patients having exposure to chemotherapy and/or external beam radiotherapy and receiving between four and seven cycles of PRRT total (Table 4). The mean latency from first PRRT to diagnostic bone marrow sampling was 54 months (range 8-132 months).

**Table 3:** Response to treatment—imaging and hr-QoL

Radiological response rates		
	RECIST 1.1 category	Number of patients
Complete iPRRT course (results available for 131/143 patients)	Partial response	19 (15%)
	Stable disease	99 (76%)
	Progressive disease	13 (10%)
Partial iPRRT course (results available for 22/39 patients)	Stable disease	15 (68%)
	Progressive disease	7 (32%)
Retreatments (results available for 47/69 cumulative rPRRT courses)	Partial response	3 (6%)
	Stable disease	33 (70%)
	Progressive disease	11 (23%)

Change in hr-QoL scores over iPRRT			
Composite domain (questions included)	Mean score (%)		*P* value when significantly different
	PRE	POST	
A: Global health score (Q29/30)	65	70	0.001
B: Physical functioning (Q1-5)	82	82	n.s
C: Emotional functioning (Q21-24)	76	79	<0.01
D: Social functioning (Q42, 44, 49)	65	72	<0.001
E: GI symptoms (Q34-38)	22	17	<0.001
F: Treatment -related symptoms (Q39, 40, 46)	13	13	n.s.
G: Disease-related worries (Q41, 43, 47)	39	32	<0.001
H: Endocrine symptoms (Q31-33)	19	17	n.s.

hr-QoL, health-related quality of life; iPRRT, induction peptide receptor radionuclide therapy; n.s., not significant; rPRRT, retreatment peptide receptor radionuclide therapy.

**Table 4:** Characteristics of the eight patients diagnosed with haematological malignancies between 2011 and 2022

Year of haematological diagnosis	Age (years) at haematological diagnosis	Gender	Haematological diagnosis	Latency period (months)^a^	Total PRRT cycles	Other bone marrow-toxic treatments received before the diagnosis of the haematological malignancy
2013	58	Female	t-MDS	8	4	1 Cycle PRCRT with 5FU associated with coronary spasm
2015	65	Female	t-MDS	34	5	Prior chemoradiotherapy for breast cancer. ERBT (20 Gy) for NET. 4 Cycles PRCRT with 5FU
2015	51	Female	t-AML	132	7	Prior EBRT (7 different courses, total Gy unknown) and chemotherapy (streptozotocin/5FU) for NET. 5 Doses of In-111 octreotide
2016	80	Male	t-MDS to AML	38	4	4 Cycles PRCRT with 5FU
2017	68	Female	t-MDS	34	4	None
2019	80	Male	t-MDS	44	4	Multiple courses of prior EBRT (120 Gy) for NET
2021	70	Female	t-MDS	49	5	Prior chemotherapy (carboplatin, etoposide) for NET
2022	78	Male	t-AML	91	7	Prior chemotherapy (5FU, dacarbazine, CAPTEM) for NET. 5 Cycles of PRCRT with 5FU

5FU, 5 fluorouracil; AML, acute myeloid leukaemia; CAPTEM, capecitabine/temozolomide; EBRT, external beam radiotherapy; Gy, Gray; NET, neuroendocrine tumour; PRCRT, peptide receptor chemoradionuclide therapy; PRRT, peptide receptor radionuclide therapy; t-MDS, treatment related myelodysplasia.

a Latency period is defined as the time from first cycle of PRRT to the bone marrow sample/diagnosis date.

Impaired renal function during therapy resulted in two patients being unable to complete a full induction course. One patient demonstrated improvement in renal function and subsequently received two cycles of rPRRT. The other progressed to end-stage renal failure due to chronic hypertension.

At the 3-month follow-up after iPRRT, eGFR results were available for 130/182 patients. The eGFR was <60 ml/min/1.73 m^2^ in 21/130 (16%).

Longer term follow-up eGFR results from within 6 months of death or census date were available for 175/189 (93%) patients. The eGFR was <60 ml/min/1.73 m^2^ in 61 (35%) patients, of whom 15 (9%) had an eGFR <30 ml/min/1.73 m^2^. Upon review of the 15 patients whose eGFR fell to <30 ml/min/1.73 m^2^, PRRT was identified as a contributor in only 1 patient. The causes of renal failure in the remaining 14 patients were sepsis (5), other acute medical illness (4), cardio- or hepatorenal syndrome (2), chronic hypertension requiring dialysis (1), obstructive uropathy due to intra-abdominal NET (1), and unknown (1).

## Discussion

This paper reports on our centre’s experience delivering a PRRT service using a clinical audit method to summarise patient characteristics, treatment, and outcomes. PRRT with ^177^Lu-DOTATATE is incorporated into multiple national^17^ and international^18^, ^19^, ^20^ NEN/NET treatment guidelines. It is currently recommended after progression on SSA therapy, however the recently published NETTER-2 trial may see PRRT increasingly used as first line in those with WHO grade 2/3 GEP-NETs.^21^ During the audit period, our model of care for both treatment and restaging followed the available guidelines at the time. The ongoing challenges in this area are well described.^22^

Concurrent chemotherapy was predominantly recommended for our patients with poor prognostic features such as high-grade disease and high tumour burden. There are increasing data on the safety and efficacy of concurrent chemotherapy.^23^^,^^24^ Larger phase III trials are underway to further define the optimal patient cohort of combination treatment due to the potential for greater haematological toxicity.

It is increasingly recognised that dual tracer imaging with FDG PET in addition to [^68^Ga] DOTATATE PET may identify patients with higher grade or heterogenous disease who would benefit from the addition of concurrent chemotherapy. FDG avidity is usually a poor prognostic indicator in a subset of patients,^25^^,^^26^ and functional scoring comparing SSTR with FDG imaging (NETPET score) can aid decision-making.^27^ Whilst not yet routinely included in PET reports, the NETPET score is manually assigned at our NET MDT.

We measured a median OS of 48.4 months (95% CI 42.5-57.3 months), which is comparable to the 48.0 months (95% CI 37.4-55.2 months) reported by NETTER-1^3^ and other single-centre publications.^28^^,^^29^ Unsurprisingly, patients who completed an induction course of at least four cycles had a higher OS compared with patients who were unable to complete a full course.

NETTER-1 excluded patients with GFR <50 ml/min, however, more recent guidelines and publications accept <40 ml/min as the cut-off for exclusion.^21^^,^^28^^,^^30^^,^^31^ All our patients met eligibility based on GFR >40 ml/min derived from DTPA nuclear medicine renal perfusion study, rather than the calculated eGFR. The latter was used for follow-up and assessment of renal toxicity. As we did not routinely repeat the DTPA study after completion of PRRT, a more detailed statistical analysis of renal toxicity was not possible. Additionally, many laboratories historically reported eGFR values as ‘>60 ml/min/1.73 m^2^’ instead of providing an absolute value. The proportion of patients with eGFR <60 ml/min/1.73 m^2^ at 3 months was similar to baseline (13% versus 16%), however, this proportion increased to 35% at longer follow-up or death. PRRT was not considered the leading cause of declining renal function in patients with eGFR <30 ml/min/1.73 m^2^ at death or census date. With an increasing number of long-term cancer survivors, we acknowledge this cohort of patients are at risk of multifactorial renal decline due to increasing age, comorbidities, and cumulative toxicities from a range of cancer therapies including PRRT. DTPA renal perfusion studies could be incorporated into future studies evaluating delayed onset renal toxicity.

Our rate of severe grade 3/4 haematological toxicity (4.2%) is comparable to other centres and publications, which range from 2.2% to 7.0%.^28^, ^29^, ^30^, ^31^ Most of these patients had also received chemotherapy and/or radiotherapy. Since the introduction of the REDCap™ database in late 2021, we have routinely recorded all haematological and biochemical results to allow future analyses of all toxicities as per Common Terminology Criteria for Adverse Events (CTCAE) v5.0 grading. We will be able to report on all CTCAE grades in future studies. Additionally, we now routinely record the results of echocardiogram and/or N-terminal pro-brain natriuretic peptide before PRRT to assess the prevalence of carcinoid heart disease. In combination with clinical symptoms and biomarkers, this cardiac specific information is used to risk stratify patients at increased risk of PRRT-induced carcinoid crisis.

We have demonstrated that treatment with PRRT resulted in statistically significant improvements in hr-QoL across multiple domains. It is well known that navigating a complex health system with an uncommon cancer causes anxiety, time, and financial toxicity. When combined with a high symptom burden due to hormone excess, hr-QoL is often significantly impacted for patients with metastatic SSTR-expressing malignancies. The early adoption and integration of hr-QoL surveys as part of our routine care pathway has translated to a greater understanding of patient well-being, perceptions towards health, and challenges for rural and remote patients.

### Limitations

There are several limitations to our data including their retrospective nature and personalisation of treatment regimens within the available protocols that need to be taken into account when comparing our findings with other services.

We did not evaluate the time from MDT recommendation to commencing PRRT. This was contingent on many factors including clinical need, stabilisation of other comorbidities and acute medical episodes, organising logistics for rural and remote patients, radiopharmaceutical supply chain, and service availability.

We relied on predominantly structural imaging when gathering treatment response data. RECIST criteria were not routinely reported, and this was retrospectively evaluated. Choice of follow-up imaging was at the discretion of the primary treating clinician, thus some patients underwent structural imaging alone (contrast enhanced CT), whereas others underwent functional imaging ([^68^Ga] DOTATATE PET ± FDG PET). Although the additive role of functional imaging is well established, there was limited availability for our cohort. The use of CT to restage aligns with the latest Australian^17^ and European guidelines.^17^^,^^18^

After the 3-month post-treatment review, ongoing care was provided by the primary treating clinician, and follow-up data were not always available.

### Conclusions

We have presented our single-centre experience over an 11-year period from a complex and heterogenous patient cohort. Our findings are consistent with other published data demonstrating safety and efficacy of ^177^Lu-DOTATATE for metastatic or locally advanced SSTR-expressing malignancies. We have demonstrated that treatment with PRRT resulted in statistically significant improvements in hr-QoL across multiple domains. We advocate for routinely evaluating hr-QoL indices to guide clinical care and ultimately improve patient’s outcomes and quality of life.
